# Supplementary figures and images for: Human IFITM3 restricts chikungunya virus and Mayaro virus infection and is susceptible to virus-mediated counteraction
Source: Life Sci Alliance. 2021 Jun 2;4(7):e202000909. doi: 10.26508/lsa.202000909 (PMC8200292; doi:10.26508/lsa.202000909)

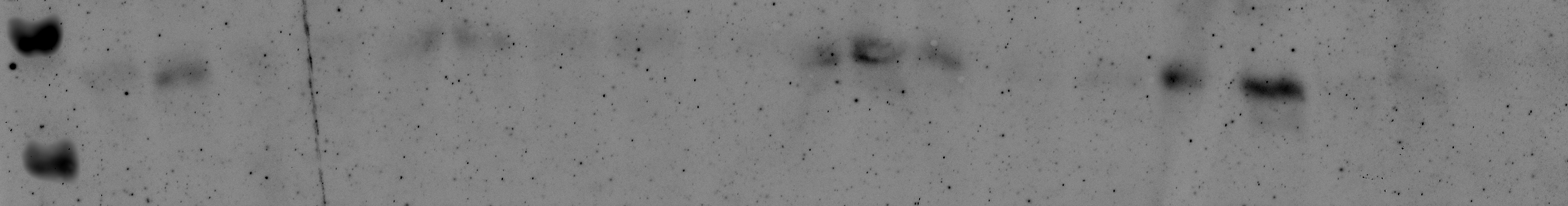

Supplement: Supplementary file 1 [file LSA-2020-00909_SdataF1.zip › Fig. 1A/20180923 HeLa IFITM2 blot anti IFITM2.png]

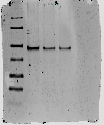

Supplement: Supplementary file 1 [file LSA-2020-00909_SdataF1.zip › Fig. 4C/Vector anti-Actin/0000958_04_TH.jpg]

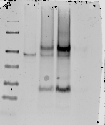

Supplement: Supplementary file 1 [file LSA-2020-00909_SdataF1.zip › Fig. 4C/Vector anti-CHIKV/0000964_06_TH.jpg]

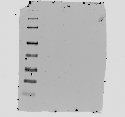

Supplement: Supplementary file 1 [file LSA-2020-00909_SdataF1.zip › Fig. 4C/Vector anti-HA/0000953_03_TH.jpg]

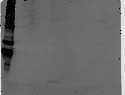

Supplement: Supplementary file 1 [file LSA-2020-00909_SdataF1.zip › Fig. 6A/Hela supernatant_anti-HIV p24/0000832_04_TH.jpg]

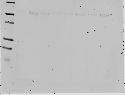

Supplement: Supplementary file 1 [file LSA-2020-00909_SdataF1.zip › Fig. 6A/Hela superntant_anti-IFITM3/0000827_04_TH.jpg]
